# Compensation Method Based on Phase Shift Between Pins of Crystal Resonator

**DOI:** 10.3390/s25030932

**Published:** 2025-02-04

**Authors:** Zhiqi Li, Jiale Peng, Miao Miao, Zicong Wang

**Affiliations:** School of Mechano-Electronic Engineering, Xidian University, Xi’an 710071, China; 22041212724@stu.xidian.edu.cn (J.P.); mmiao@mail.xidian.edu.cn (M.M.); 22041212659@stu.xidian.edu.cn (Z.W.)

**Keywords:** OCXO (oven-controlled crystal oscillator), resonance parameters, frequency drift, phase shift, self-calibration

## Abstract

This paper presents a new method to improve the long-term frequency stability of an oven-controlled crystal oscillator (OCXO) without an external reference source. The frequency drift of the crystal oscillator can be obtained in real time by measuring the phase shift between the pins of the crystal resonator. In this paper, according to an equivalent circuit of a crystal oscillator, a linear equivalent mathematical model based on the phase shift of the crystal resonator and the output frequency is established. The experiments were conducted to observe the relation between the phase shift and frequency drift of the OCXO. At the same time, a crystal oscillator self-calibration frequency control system is established to improve the frequency drift of the OCXO. The results show that this method can effectively improve the drift of OCXOs. The OCXO drift rate was significantly improved from 1.53 10^−10^/day to 9.8 10^−12^/day, and after three days, it settled at 5.24 10^−11^. The long-term frequency stability also underwent remarkable improvement, from 1.16 10^−11^/1000 and 3.24 10^−11^/10,000 to 3.47 10^−12^/1000 and 1.05 10^−11^/10,000.

## 1. Introduction

Crystal oscillators have been widely utilized in various measurement instruments, navigation, and space exploration, attributed to their high frequency stability and relatively low cost. Because of its poor daily aging or long-term frequency stability, the crystal oscillator cannot serve as an independent reference source for high-accuracy fields such as time services, time keeping, and 5G communication base stations. For instance, in the high-performance OCXO OSA8607, Rakon’s HSO14, daily aging can only achieve ±0.5 ppb; the effective solution is to lock the OCXO with an additional highly accurate frequency reference such as an atomic clock, GNSS signals, and so on [1,2,3].

Research on the frequency aging estimation or compensation of OCXOs without a reference source has always been a hotspot in the field of time and frequency. As early as 1993, Raymond L. Filler concluded that the logarithmic model can reflect the aging trend of OCXOs. A mathematical model is established using the aging data of 30 days. However, in most cases, this model cannot effectively predict the one-year aging of the crystal oscillator [4]. Professor Zhou Wei used the nonlinear least squares method to establish the parameter estimation and aging drift curve of the model according to the data of the first 30 days [5]. Although these models can reflect the trend of crystal oscillator frequency drift, the actual compensation effect for OCXO aging is not very satisfying. The aging data of crystal oscillators is very discrete, and crystal oscillators exhibit different aging characteristics at different stages. The aging estimation method is still a possible way to solve OCXO frequency drift over a long time period and has been widely researched [6,7,8]. Ref. [9] adaptively simulates the frequency drift characteristics of base station time reference OCXOs by locking to the satellite time reference signal. If the satellite signal is lost within holding period 24 h and the time offset is less than 1.5 us, it means that the aging rate is improved from 3.4 10^−10^/d to 1.7 10^−11^/d. But after 24 h, the frequency drift increases rapidly as time goes by. Ref. [10] used the PSO-BP neural network model to fit and predict the aging data of two groups of crystal oscillators. The system successfully maintained the frequency accuracy within ±8 10^−11^ during the 24 h after the loss of the external high-accuracy reference source. The aging rate of the OCXO was improved from 5 10^−10^/d to less than 8 10^−11^/d. During the data acquisition process, it is difficult to separate the effects of other factors from the output frequency. Therefore, aging estimation methods make a very limited improvement on the calibration of the output frequency of OCXOs, and the effectiveness gradually deteriorates with time. It is generally only suitable for the short term, within 24 h or less. These models can reflect the trend of frequency drift in crystal oscillators, yet crystal oscillators exhibit different aging characteristics at various stages. The effectiveness of aging estimation methods in improving the OCXO is limited and gradually deteriorates with time, typically being suitable for periods of 24 h or several hours only, which are only suitable for clock keeping after being confirmed with an external reference [11]. In contrast, our method can reduce the drift of OCXO output frequency to ±6 10^−11^ within three days, and the aging rate is improved from 1.53 10^−10^/day to 9.8 10^−12^/day, and 4.59 10^−10^/3 days to 5.24 10^−11^/3 days.

Generally, a crystal resonator can be considered equivalent to a resonant circuit that comprises inductors, capacitors, and resistors. This equivalence enables the impacts of aging and various other factors to be accurately reflected in the corresponding parameters of the crystal resonator [12]. Theoretically, measuring the equivalent resonator parameters can be used as a method to determine the frequency drift of crystal oscillators. However, for high-performance crystal oscillators, their daily aging is better than 0.5 ppb. The equivalent resonance parameters themselves are very small, and with smaller variances, they are difficult to measure. High-precision crystal impedance meters and vector network analyzers are effective means, but they cannot be used for real-time frequency drift detection and compensation in OCXOs [13].

This work introduces a novel method for obtaining the frequency drift in an OCXO without an external reference source. The method involves monitoring the resonator’s phase shift in real time, which has not been previously reported in the literature. We derive a linear equation relating the phase shift between the crystal resonator pins to its frequency. It is demonstrated that the frequency drift can be accurately reflected in the phase shift. By conducting real-time phase measurements, the frequency drift can be controlled or determined based on the phase shifts observed under dynamic conditions, unlike the available methods, which use external high-accuracy frequency sources or use aging data for prediction. The main contributions of this paper are as follows:

(1) Studying the working process of an OCXO, a mathematical model of the phase shift between the crystal resonator pins and the OCXO’s frequency is established. It is proved that the frequency drift can be reflected in the phase change.
(2) Through the real-time measurement of phase, it is found that the phase shift and frequency drift can be equivalent to a linear relationship.
(3) Combined with characteristics of the OCXO and digital phase identification technology, the frequency drift can be controlled according to the phase shift obtained under dynamic conditions. Frequency drift and the long-term stability are improved.

## 2. Crystal Resonator Phase Shift and Frequency Drift

**Figure 1:**
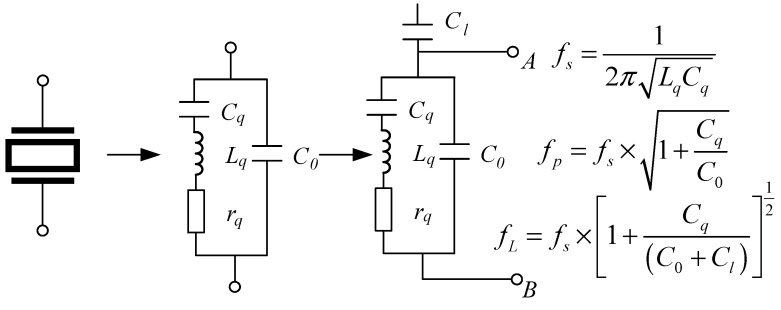
The equivalent circuit model of the crystal resonator after it is connected to the oscillation circuit, where $C_{l}$ is the equivalent load capacitance of the peripheral circuit, and the frequency of the circuit is $f_{L}$ ($f_{s}$ < $f_{L}$ < $f_{p}$).

A quartz crystal resonator can be seen as a mechanical and electrical energy conversion device that can be equivalent to a combination of capacitive and inductive elements in a circuit, and its piezoelectric effect can be represented by the equivalent circuit shown in Figure 1 [14,15]. $C_{0}$, $C_{q}$, $L_{q}$, and $r_{q}$ are the equivalent resonant parameters. $C_{0}$ is the static capacitance between the two plates of the resonator, which mainly includes the capacitance formed between the electrodes and the parasitic capacitance of the crystal and the shell; $L_{q}$ is the dynamic inductance; $C_{q}$ is the dynamic capacitance, which represents the rigidity and elasticity of the quartz crystal, which is related to the electrode area, thickness, and shape. This value is relatively small; $r_{q}$ is the dynamic resistance of frictional loss during vibration [16,17].

The circuit, composed of capacitance and inductance, has a phase shift effect. When the parameter $L_{q}$ or $C_{q}$ varies, it will cause a relative variation in the impedance angle. In this circuit, the effect of the peripheral circuitry on the resonator can be equivalent to a load capacitance $C_{l}$ connected in series with the equivalent circuit. The frequency of the circuit is $f_{L}$ ($f_{s}$ < $f_{L}$ < $f_{p}$). The reactance X is as follows:

(1) $$X=\frac{\omega^{2}L_{q}C_{q}-1}{\omega \left(C_{q}+C_{0}-\omega^{2}L_{q}C_{q}C_{0}\right)}$$

where ω is the resonant angular frequency. It can be obtained when the crystal oscillator is series resonant, X=0, and the frequency is the series resonant frequency $f_{s}$:

(2) $$\left\{\begin{array}{l} \omega_{s}=\frac{1}{\sqrt{L_{q}C_{q}}}\\ f_{s}=\frac{1}{2\pi \sqrt{L_{q}C_{q}}} \end{array}\right.$$

When the crystal oscillator is resonant in parallel X→∞ and the frequency is the parallel resonant frequency $f_{p}$:

(3) $$\left\{\begin{array}{l} \omega_{p}=\frac{1}{\sqrt{L_{q}C_{q}}}\sqrt{1+\frac{C_{q}}{C_{0}}}\\ f_{p}=\frac{1}{2\pi \sqrt{L_{q}C_{q}}}\sqrt{1+\frac{C_{q}}{C_{0}}} \end{array}\right.$$

The impedance angle is as follows:

(4) $$\varphi =arctan\frac{\omega^{2}L_{q}C_{q}-1}{\omega r_{q}(C_{q}+C_{0}-\omega^{2}L_{q}C_{q}C_{0})}$$

where ω is the angular frequency; $r_{q}$ is the dynamic resistance. The circuit composed of capacitance and inductance has a phase shift effect. When the parameters $L_{q}$ and $C_{q}$ change, it causes a synchronous change in the impedance angle. When an oscillation circuit is applied to the crystal resonator, the effect of the peripheral circuitry on the resonator can be equivalent to a load capacitance $C_{l}$ connected in series with the equivalent circuit.

A working crystal resonator is in parallel resonance, and its output frequency is expressed as

(5) $$f_{L}=f_{s}\times {\left[1+\frac{C_{q}}{\left(C_{0}+C_{l}\right)}\right]}^{\frac{1}{2}}$$

The impedance angle φ can be defined as

(6) $$\left\{\begin{array}{l} \varphi =arctan\frac{T_{s}}{G_{p}r_{q}}\\ T_{s}=\omega^{2}L_{q}C_{q}-1\\ G_{p}=\omega \left(C_{q}+C_{0}-\omega^{2}L_{q}C_{q}C_{0}\right) \end{array}\right.$$

When the crystal oscillator circuit is in an inductive state, the inductance $L_{q}$ plays a major role. If $L_{q}$ varies by $\Delta L_{q}$, the impedance angle φ and the frequency $f_{L}$ vary by Δφ and $\Delta f_{L}$, respectively:

(7) $$\left\{\begin{array}{l} \frac{\Delta \varphi }{\Delta L_{q}}=\frac{r_{q}}{r_{q}^{2}G_{p}^{2}+T_{s}^{2}}\left(\frac{\partial T_{s}}{\partial L_{q}}G_{p}-\frac{\partial G_{p}}{\partial L_{q}}T_{s}\right)\\ \frac{\Delta f_{L}}{\Delta L_{q}}=-\frac{1}{4\pi \sqrt{C_{q}L_{q}^{3}}}\sqrt{1+\frac{C_{q}}{C_{0}+C_{l}}} \end{array}\right.$$

then:

(8) $$\Delta \varphi =-\frac{4\pi \omega_{L}^{2}L_{q}r_{q}{\left(C_{0}+C_{l}\right)}^{2}}{r_{q}^{2}\omega_{L}^{2}C_{l}^{2}+1}\Delta f_{L}$$

where $\omega_{L}=2\pi f_{L}$; $\Delta \varphi /\Delta f_{L}$ can be defined as k:

(9) $$k=-\frac{4\pi \omega_{L}^{2}L_{q}r_{q}{\left(C_{0}+C_{l}\right)}^{2}}{r_{q}^{2}\omega_{L}^{2}C_{l}^{2}+1}\;(\mathrm{rad}/\mathrm{Hz})$$

For a 10 MHz OCXO, if $C_{l}$ is around one hundred picofarads, $r_{q}$ is about tens of ohms. With different initial angular frequencies $\omega_{L1}$ and $\omega_{L2}$, there exist $k_{1}$ and $k_{2}$; $r_{q}^{2}\omega_{L1}^{2}C_{l}^{2}$ and $r_{q}^{2}\omega_{L2}^{2}C_{l}^{2}$ are far less than 1; Δk can be expressed as:

(10) $$\Delta k\approx -4\pi \left(L_{q1}-L_{q2}\right)r_{q}{\left(C_{0}+C_{l}\right)}^{2}\left[\omega_{L1}^{2}-\omega_{L2}^{2}\right]$$

where $L_{q1}$ and $L_{q2}$ are dynamic inductance for $\omega_{L1}$ and $\omega_{L2}$.

Frequency drift caused by aging for a high-performance OCXO is far less than 1 Hz in the short term, $\omega_{L1}-\omega_{L2}\ll 2\pi \;\mathrm{rad}/s$. It can be concluded that $\Delta k\ll 10^{-4}$. Compared to k itself, the variation Δk is very small. k can be approximated as a constant, which denotes the conversion coefficient. There is a linear relationship between the impedance angle variation Δφ and the output frequency variation $\Delta f_{L}$.

According to $\Delta \theta =\Delta \varphi /\left(2\pi f_{L}\right)$, the angle variation Δφ can be converted to the phase shift Δθ in second, and the phase shift is easier to measure than the varies of the equivalent inductance parameter. Here, we take the experimental OCXO as the example. The static capacitance $C_{0}$ is 2.38 pF, the dynamic capacitance $C_{q}$ is about 0.183 fF, the dynamic resistance $r_{q}$ is 69 Ω, and the initial value of the dynamic inductance $L_{q}$ is 1387 mH. When the load capacitance $C_{l}$ is 120 pF, $f_{L}=9.990\times 10^{6}\;\mathrm{Hz}$, and by Equation (7) we can obtain

(11) $$\left\{\begin{array}{l} \Delta \varphi =3.52926\times 10^{6}\Delta L_{q}\\ \Delta f_{L}=-3.60124\times 10^{6}\Delta L_{q} \end{array}\right.$$

The unit of the phase difference Δφ is in rad, which is converted into a phase shift Δθ in seconds:

(12) $$\;\left\{\begin{array}{l} \Delta \theta =5.62270\times 10^{6}\Delta L_{q}\\ \Delta f_{L}=-3.60124\times 10^{6}\Delta L_{q} \end{array}\right.$$

From Equation (12), when the dynamic inductor $L_{q}$ increases by 1 nH, the output frequency decreases by about 3.6 10^−3^ Hz, and the phase of the signal increases by about 4.4 10^−3^ ns.

**Figure 2:**
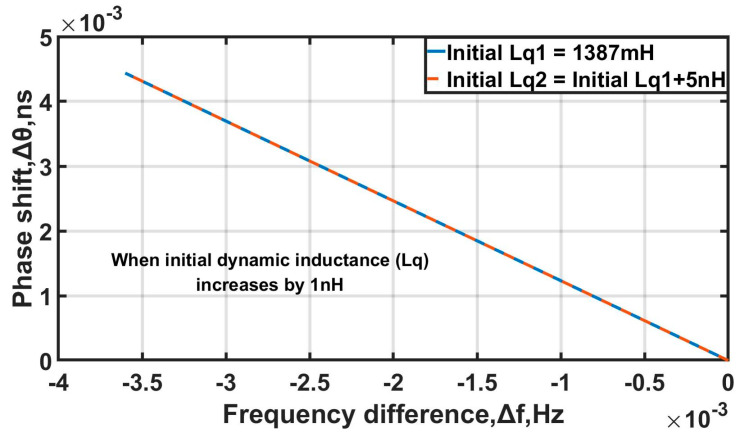
Frequency difference vs. phase shift.

The daily aging rate of a common high-performance OCXO does not exceed 1 10^−9^, which means that the equivalent inductance parameter changes do not exceed 5 nH. When the equivalent inductance parameter increases by 5 nH, the conversion factor k=−1.232. k only changes −3.539 10^−9^ in Figure 2, which causes a very small error that can be ignored. So, k can be seen as a constant under the conditions.

**Figure 3:**
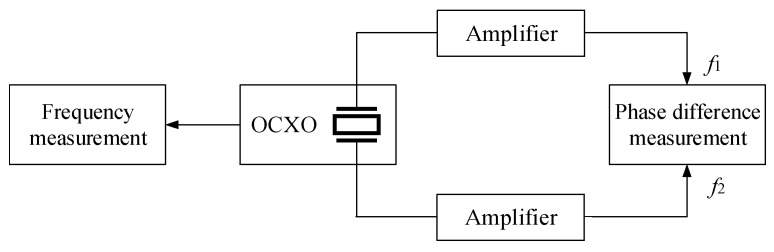
Block diagram of phase shift and frequency measurement experiment: the two pins of the crystal resonator are drawn out, and the outputs $f_{1}$ and $f_{2}$ are connected to 53230A to measure the phase shift.

**Figure 4:**
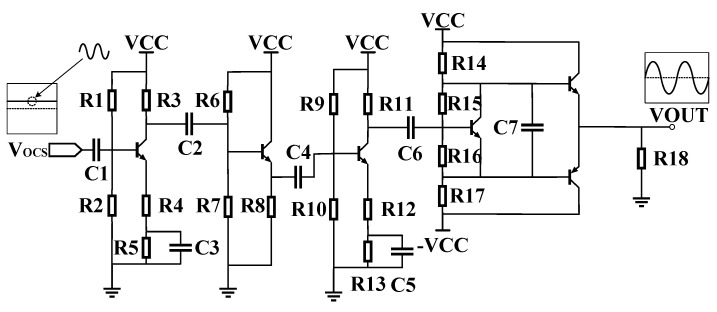
Power amplification circuit.

The block diagram of the verification experiment for the relationship between frequency and phase is shown in Figure 3. The frequency of the OCXO and the phase shift of the internal resonator were simultaneously acquired. The 10 MHz OC25-QYJ09 crystal oscillator was selected as the experimental OCXO, whose frequency stability is ≤1 10^−11^/s, daily aging is ≤0.5 ppb, and temperature drift is ≤5 ppb. The Microsemi 3120A was used to measure the frequency of crystal resonator. The phase shift between the two pins of the resonator was measured using a Keysight Technologies 53230A. The TR2001 rubidium atomic standard was used as the frequency reference. The signals from the resonator need to be amplified for measuring. In particular, one of the signals is very weak and is customarily ignored. We designed a signal amplifier as in Figure 4. In the experiment, the sampling interval of 53230A and 3120A was set to 0.1 s, and the room temperature was 23~25 °C.

**Figure 5:**
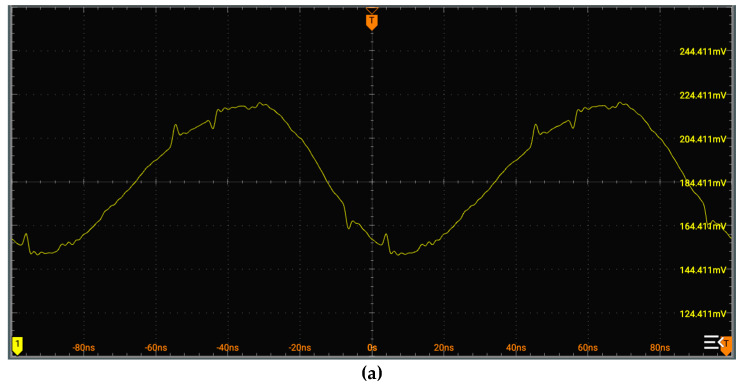

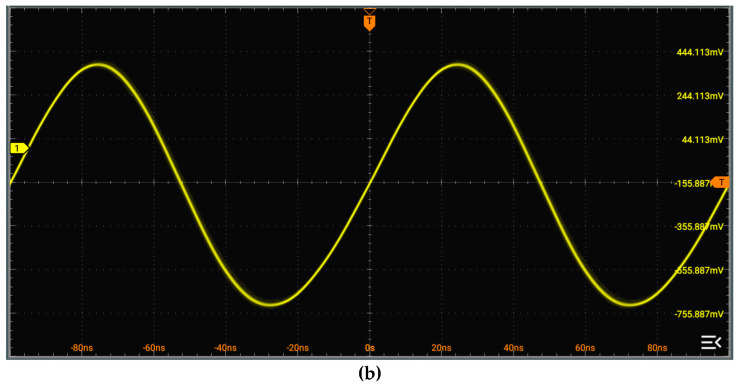
Comparison before and after signal amplification: (**a**) signal before amplification; (**b**) signal after amplification.

In Figure 4, the amplifying circuit in the figure consists of two common-emitter amplifier circuits, an emitter follower circuit, and a push–pull output circuit. The sine signal from resonator is in the several tens of millivolts with DC bias. After amplification, the signals can provide phase detection. For instance, the waveform of signal $f_{1}$ captured on a high-resolution oscilloscope is shown in Figure 5, with a peak-to-peak voltage of approximately 60 mV. After amplification, the peak-to-peak voltage reaches about 800 mV.

**Figure 6:**
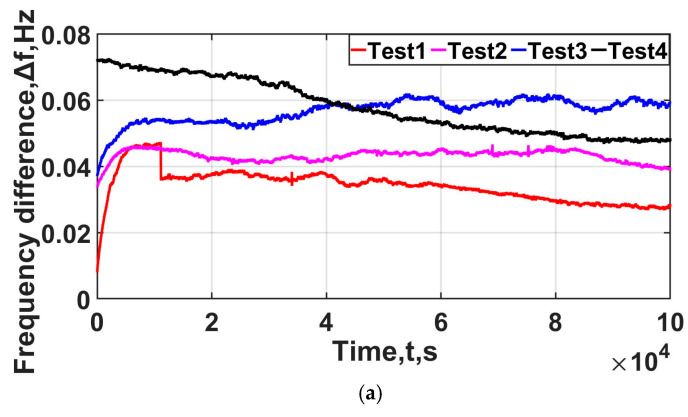

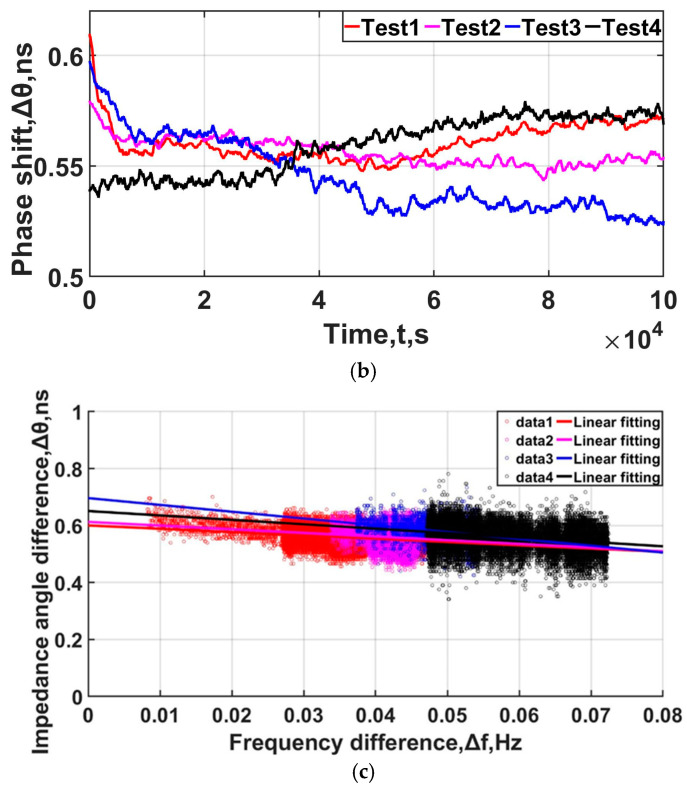
Measurement data of phase and frequency: (**a**) Frequency drift in the OC25-QYJ09; the unit is hertz, and its initial value $f_{0}$ is 9.99998242 MHz. (**b**) The phase shift between pins of the resonator, in nanoseconds; its initial value $f_{0}$ is 41.6 ns. (**c**) Frequency difference vs. phase shift.

The experiment was conducted four times; the results of Tests (1–4) for the OC25-QYJ09 are shown in Figure 6. Figure 6a shows the frequency drift of the OCXO over time in four experiments, each lasting for 3 h. It can be observed that the frequency drift of the crystal oscillator over time varies among the experiments; there is a variation of approximately 1–4 ppb, roughly speaking. Figure 6b displays the phase shift between pins of the resonator over time in these four experiments, with each experiment having a variation within 0.1 ns. Figure 6c shows the relationship between the frequency drift of the OCXO and the phase difference between the pins of the crystal resonator in the four experiments, revealing a linear correlation between them. The four measured values are, respectively, k1 = −1.157, k2 = −1.289, k3 = −2.388, and k4 = −1.548. From Figure 6, we can see that the law of frequency data is different, and it cannot easily be expressed linearly or logarithmically. The frequency difference and phase shift in the four tests show a linear relationship. When the frequency increases by 0.1 Hz, the phase decreases by about 0.1 ns. We used the least squares method for fitting, where the linearity of data (1–4) is 0.9457, 0.9926, 0.8707, and 0.9055. Finally, the slope of data1 and data2 were selected to calculate the measured k value, which is −1.187.

## 3. Experiment and Discussion

**Figure 7:**
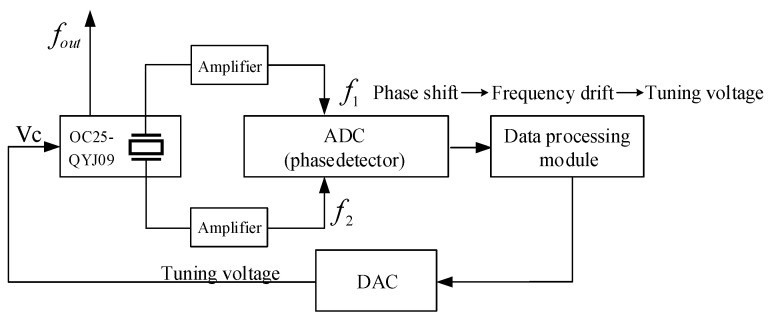
Block diagram of the self-calibration system. The crystal oscillator OC25-QYJ09 is used as the sample crystal oscillator, $f_{1}$ and $f_{2}$ are the outputs of the crystal oscillator resonator, and $f_{out}$ is the output frequency after calibration. The system uses an analog-to-digital converter (ADC) as the phase detector to acquisition of phase data. The data processing module adopts an STM32 microcomputer and DAC to achieve feedback control.

The block diagram of the self-calibration system of controlling the output frequency by the real-time measurement phase is shown in Figure 7.

In Figure 7, the system uses a 16 bit analog-to-digital converter (ADC) as the phase detector. The output signal $A\left(t\right)$ of the crystal oscillator can be expressed as

(13) $$A(t)=A_{m}\sin\left(2\pi f_{0}t+\varphi \right)$$

In Equation (13), $A_{m}$ is the peak output voltage, $f_{0}$ is the nominal frequency, and φ is the phase. As shown in Figure 7, the zero-crossing of the rising edge of $f_{1}$ is used as the trigger point for ADC sampling; assuming that the acquired amplitude is $A_{i}$, the phase variation Δφ can be calculated by Equation (14), and frequency drift of the OCXO can be obtained.

(14) $$\Delta \varphi_{i}=\varphi_{i+1}-\varphi_{i}=\frac{\arcsin\frac{A_{i+1}}{A_{m}}-\arcsin\frac{A_{i}}{A_{m}}}{2\pi f_{0}}$$

where $A_{i}$ and $A_{i+1}$ are the acquired amplitudes. By modulating the tuning voltage feedback to the voltage control terminal of the crystal oscillator through DAC step output, frequency drift can be improved [18]. To mitigate the effects of the OCXO’s startup characteristics and allow for it to work stably, it is essential to let the OCXO operate for 10 h prior to performing the experiment. Additionally, the room temperature should be maintained between 23 °C and 25 °C.

**Figure 8:**
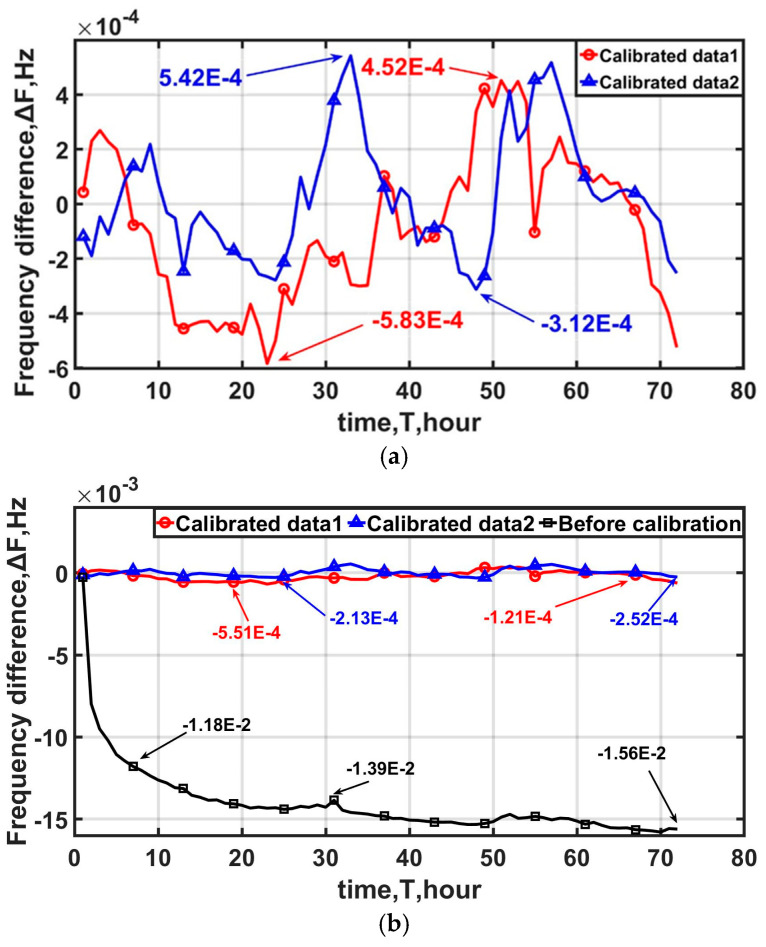
The frequency data before and after calibration: (**a**) the frequency fluctuates back and forth in the range of ±6 10^−4^ Hz; (**b**) the aging rate was reduced from 4.59 10^−10^/3 days to 5.24 10^−11^/3 days.

After calibration, the frequency fluctuation is within ±6 10^−4^ Hz, and the daily aging rate can reach 9.8 10^−12^/d by linear fitting. In Figure 8, the aging rate was reduced from 1.38 10^−9^ to 5.24 10^−11^ after 3 days.

**Figure 9:**
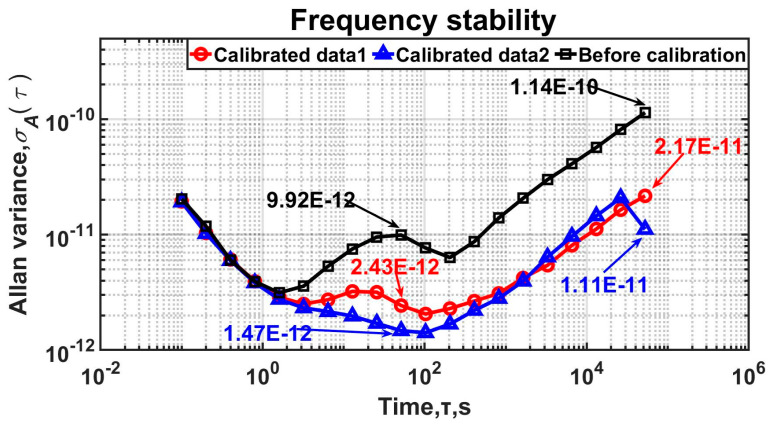
Allan variance before and after calibration. The aging rate was reduced from 4.59 10^−10^/3 day to 5.24 10^−11^/3 day. The frequency stability of 10,000 s after calibration is 1.05 10^−11^ and 1.26 10^−11^, which is an improvement from 3.24 10^−11^.

In Figure 9, the frequency stability starts to improve after 1 s; in particular, at 1000 s, it enhances by about one order of magnitude. It is obvious that adoption of the method in this paper significantly improves the long-term frequency stability of the oven-controlled crystal oscillator (OCXO) without the need for an external reference source. Detailed data on the long-term frequency stability are shown in Table 1. The frequency stability is improved overall. In particular, the frequency stability at the 100 s averaging time improves from 1.16 10^−11^ before calibration to 3.47 10^−12^ and 2.96 10^−12^.

## 4. Conclusions

The research presented in this paper introduces a novel methodology designed to improve frequency drift in OCXOs. Frequency drift in OCXOs can be deduced from variations in the resonance parameters of the crystal resonator, as well as the phase shift observed at both pins of the resonator. Specifically, for the sample OCXO, OC25-QYJ09, when the resonance parameters of the crystal oscillator, such as the equivalent inductance, changes approximately 0.3 nH, the frequency drift is approximately 1 10^−3^ Hz. Correspondingly, the phase shift of the crystal resonator changes by 1 10^−3^ ns, which can be accurately measured by an ADC. By continuously monitoring these phase shifts in real time, the issue of frequency drift compensation in OCXOs is effectively addressed without an external reference source, thereby significantly enhancing its operational performance. Furthermore, through a self-calibration system experiment, the aging effect on OCXOs is effectively improved by nearly three orders of magnitude within a period of 3 days, all without the reliance on external reference sources.

## Figures and Tables

**Table 1:** Frequency stability before and after calibration measured by 3120A.

τ	Frequency Stability (σ)		
	Before Calibration	Calibrated Data1	Calibrated Data2
1 s	3.55 10^−12^	3.51 10^−12^	3.38 10^−12^
10 s	6.75 10^−12^	3.05 10^−12^	2.05 10^−12^
100 s	7.54 10^−12^	2.09 10^−12^	1.42 10^−12^
100 s	1.16 10^−11^	3.47 10^−12^	2.96 10^−12^
1000 s	3.24 10^−11^	1.05 10^−11^	1.26 10^−11^

## Data Availability

Data will be made available on request.
